# Optimizing a Bayesian hierarchical adaptive platform trial design for stroke patients

**DOI:** 10.1186/s13063-022-06664-4

**Published:** 2022-09-06

**Authors:** Guangyi Gao, Byron J. Gajewski, Jo Wick, Jonathan Beall, Jeffrey L. Saver, Caitlyn Meinzer, the STEP-STONE Executive Committee, Colin Derdeyn, David Fiorella, Tudor Jovin, Pooja Khatri, Eva Mistry, J. Mocco, Raul Nogueira, Adnan Siddiqui

**Affiliations:** 1 Department of Biostatistics & Data Science, University of Kansas Medical Center, Kansas City, KS 66160 USA; 2 Department of Public Health Sciences, Medical University of South Carolina, Charleston, SC 29425 USA; 3 Department of Neurology and Comprehensive Stroke Center, University of California, Los Angeles, CA 90095 USA

**Keywords:** Platform trial design, Bayesian models, Hierarchical models, Response-adaptive randomization, Beta-binomial

## Abstract

**Background:**

Platform trials are well-known for their ability to investigate multiple arms on heterogeneous patient populations and their flexibility to add/drop treatment arms due to efficacy/lack of efficacy. Because of their complexity, it is important to develop highly optimized, transparent, and rigorous designs that are cost-efficient, offer high statistical power, maximize patient benefit, and are robust to changes over time.

**Methods:**

To address these needs, we present a Bayesian platform trial design based on a beta-binomial model for binary outcomes that uses three key strategies: (1) hierarchical modeling of subgroups within treatment arms that allows for borrowing of information across subgroups, (2) utilization of response-adaptive randomization (RAR) schemes that seek a tradeoff between statistical power and patient benefit, and (3) adjustment for potential drift over time. Motivated by a proposed clinical trial that aims to find the appropriate treatment for different subgroup populations of ischemic stroke patients, extensive simulation studies were performed to validate the approach, compare different allocation rules, and study the model operating characteristics.

**Results and conclusions:**

Our proposed approach achieved high statistical power and good patient benefit and was also robust against population drift over time. Our design provided a good balance between the strengths of both the traditional RAR scheme and fixed 1:1 allocation and may be a promising choice for dichotomous outcomes trials investigating multiple subgroups.

**Supplementary Information:**

The online version contains supplementary material available at 10.1186/s13063-022-06664-4.

## Introduction

Master protocols, including umbrella, basket, and platform trials, are clinical trial designs which have received increased interest in the past few years. They simultaneously evaluate multiple drugs and/or multiple populations in multiple sub-studies and thus can accelerate the drug development process [1, 2]. Platform trials simultaneously investigate multiple treatments on multiple populations and are often referred to as “multi-arm, multi-stage” (MAMS) design trials [3–7]. This type of design allows for either a fixed number of treatments or an adaptive number of treatments by dropping and/or adding treatments during the process of the trial [8]. Compared to standalone designs, they are more efficient at identifying effective treatments for specific subpopulations and can require the enrollment of fewer subjects for specific subpopulations [8]. While they may still result in a larger overall trial, they can answer treatment questions for specific subpopulations. Basket trials and umbrella trials are subtypes of platform trials. Basket trials include a single investigational drug or device being tested on multiple diseases that share a specific biomarker or mutation [9–11]. They are often used in phase II studies with the goal to explore potential uses of a treatment or identify subpopulations in which a target treatment performs well or poorly [12]. Umbrella trials, on the other hand, compare multiple investigational drugs or devices in a single disease population [13–15]. They can identify treatments that perform well or poorly for a specific disease.

Recent studies [16–24] have shown endovascular thrombectomy (EVT) is a treatment of substantial benefit in select acute ischemic stroke patients and suggested EVT is a promising potential treatment in additional, not yet interrogated, subpopulations of acute ischemic stroke patients. Given the large difference in positive outcomes for subjects treated with EVT plus medical management versus standard medical management (MM) alone observed in these trials, significant enthusiasm exists for expanding indication to additional subgroups not yet studied, as well as evaluating whether additional synergistic interventions exist. NIH-NINDS has published a notice of special interest (NOSI) in establishing a platform with a master protocol for multi-arm, multi-stage EVT trials [25]. To respond to the NOSI, the current authors, in collaboration with a team of clinical investigators, have developed the design proposed herein, with a focus on developing a first trial for performance on the platform that studies indication expansion to additional patient subgroups. If funded, the proposed “StrokeNet ThrombEctomy Platform - STarting with OptimizatioN of Eligibility” (STEP-STONE) trial, a companion trial to the platform, is a prospective, adaptive, registry-anchored trial that compares EVT plus MM medical to standard MM treatment alone, with the goal to identify patient subpopulations which can benefit from EVT treatment. In this trial, since we expect similarities in treatment differences across all subpopulations, a Bayesian hierarchical model was used to borrow information across different subgroups within a treatment arm thus improving the trial’s efficiency. In addition, given the high efficacy observed in previous subgroups, clinicians do not have the equipoise to randomize at a fixed equal allocation; instead, response-adaptive randomization is proposed to allocate patients to the more promising treatment as supportive evidence is acquired, facilitating investigator willingness to enroll.

Bayesian methods are attractive in adaptive trials, since they allow for continuous updating of posterior decision quantities as new information becomes available and thus they facilitate adapting to information obtained as a trial progresses [8, 26–30]. Motivated by previous work [31], the Bayesian hierarchical beta-binomial model used in the STEP-STONE trial included a tuning parameter in the prior distribution of response rates that adjusts the “strength” of borrowing within treatment arms.

Response-adaptive randomization (RAR) was used in the STEP-STONE trial to maximize patient benefit throughout the trial. While traditional clinical trials use fixed allocation and usually balance sample size equally in different treatment groups to eliminate bias, RAR is a patient allocation algorithm that has been commonly used in adaptive clinical trials to alter patient randomization probabilities based on interim results obtained from the trial. Updating the patient allocation ratio during the trial allows to randomize more patients to the more beneficial treatment and thus reduces the overall number of harmful events from the clinical trial and improves individual ethics [32–36].

There are many challenges accompanying the use of RAR in clinical trials, with one major challenge being patient population parameter drift [8, 37–39]. Drift occurs when the treatment response rates change over time. Without properly adjusting for drift effects, biased estimates could be obtained thus leading to wrong conclusions in the trial [37, 40, 41]. To alleviate this problem, Angus et al. used a first-order normal dynamic linear model (NDLM) to account for treatment response rates changing over time in the REMAP-CAP platform trial [42]. Motivated by their work, a drift parameter was also incorporated in the design of the STEP-STONE trial to capture the change in treatment response rates.

Another potential problem that arises in complex designs independently from RAR is the multiplicity issue. Multiplicity concerns arise when multiple comparison objectives are being evaluated in the same clinical trial [43] and failing to account for multiplicity results in inflation of type 1 errors. In the STEP-STONE trial, multiplicity occurred since multiple patient populations were included and multiple interim analyses were performed. In practice, controlling of familywise type 1 error in Bayesian designs often relies on simulation [8, 38, 44, 45]. In the STEP-STONE study, thresholds of parameters were determined to ensure overall type 1 error being controlled at 0.05 level through extensive simulation studies.

The STEP-STONE trial is a two-arm, response-adaptive platform trial. Previous research has shown [39, 46–48] that in the two-arm trial setting, compared with equal allocation, response-adaptive allocation achieved lower statistical power due to unequal sample sizes [49]. To find a compromise between high statistical power in equal allocation and the high patient benefit obtained from response-adaptive allocation, an innovative RAR scheme, “RARCOMP,” is proposed.

In summary, to address the needs of the STEP-STONE trial, we proposed a two-arm adaptive platform trial design. Our approach has three distinct characteristics: (1) the use of a Bayesian hierarchical model that allows to gain efficiency by borrowing information between subgroups, (2) an innovative RAR allocation scheme (RARCOMP) that achieves a good balance between statistical power and patient benefit, and (3) robustness to changes in the response over time. While covering all details and issues relating to platform trials is beyond the scope of this paper, the viability of our approach in two-arm trials with multiple subgroups and binary primary endpoints is demonstrated via extensive simulation studies and the proposed RARCOMP scheme could be easily adapted to multi-arm settings.

## Methods

### Motivating trial

In the STEP-STONE trial, the primary endpoint is binary and denotes if a favorable global disability level was observed at 90 days. Favorable outcome is assessed using prognosis-adjusted, sliding dichotomy analysis of the modified Rankin scale (mRS) [50–52]. Information obtained from both a prospective registry and previous related populations level I evidence trials is used to conduct patient allocation for each patient subpopulation. During the STEP-STONE trial, the patient allocation ratio is adaptively updated based on patients’ treatment responses at each interim. Once a prespecified success criterion is identified for a subgroup, all future participants in that subgroup will be assigned to the superior treatment.

#### Patient subgroups

The STEP-STONE trial will target three previously under-studied patient characteristics including (1) individuals with large ischemic cores, (2) individuals with mild deficits, and (3) individuals with distal vessel occlusions. A *large ischemic core* is defined as a substantial amount of already-injured brain tissue visualized using neuroimaging; a *mild deficit* is defined as few impairments in cognition, strength, vision, and other neurologic functions quantified using the National Institute of Health Stroke Scale; finally, a *distal vessel occlusion* refers to those strokes in which the causative clot(s) are located in intermediate (rather than large) diameter brain arteries. Of note, these characteristics are not mutually exclusive, though some combinations are clinically rare or highly unlikely, e.g., a large area of injury but only resulting in mild deficits (large ischemic core + mild deficit).

**Fig. 1:**
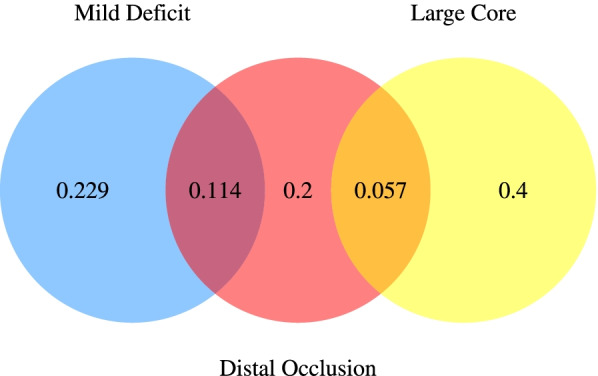
Subgroup proportion summary

Depending on whether these characteristics are present or not, patients are grouped into five mutually exclusive subgroups, which are Large Core Only, Mild Deficit Only, Distal Occlusion Only, Large core + Distal, and Mild Deficit + Distal. Figure 1 shows the Venn diagram of the five patient subpopulations and their respective expected population proportion.

### Models

Three different models will be discussed in this section. We will first start with the simplest model, the Bayesian logistic independent model in the “Bayesian logistic independent model” section, as it is a commonly used standard model for binary outcomes, and it is also the standard model in the Fixed and Adaptive Clinical Trials Simulator (FACTS) software. We then compare this model with two Bayesian hierarchical models. In the “Bayesian hierarchical model” section, we present a Bayesian hierarchical beta-binomial model that allows for borrowing information across patient subgroups within each treatment. A modified Bayesian hierarchical beta-binomial model which also accounts for patient response rates drift will be discussed in detail in the “Bayesian hierarchical drift model” section.

In all models, each of the five occurring combinations of the three patient characteristics (Mild Deficit, Distal Occlusion, Large Core) is treated as a unique subgroup without specific consideration for the base characteristics themselves. For each patient population subgroup *j* = 1, …, *J* and each treatment *k* = 1, …, *K*, where in the STEP-STONE trial *J* = 5 and *K* = 2, the number of favorable outcomes *Y*_*jk*_ follows a binomial distribution, with parameters *n*_*jk*_ and *P*_*jk*_. *n*_*jk*_, where *n*_*jk*_ represents number of participants and *P*_*jk*_ is the probability of obtaining a favorable outcome. We will introduce the three Bayesian models accordingly.

#### Bayesian logistic independent model

For the Bayesian logistic independent model, the response rates of subgroups are modeled separately each with its own prior in the independent model. In the STEP-STONE project, we assume the log odds of the response rate in each subgroup and each treatment arm follows a non-informative prior normal distribution. The complete model can be written as:

$$\log \left(\frac{P_{jk}}{1-{P}_{jk}\ }\right)={\beta}_{jk}$$

which means

$${P}_{jk}=\Pr \left( favorable\ outcomes\right)=\frac{e^{\beta_{jk}}}{1+{e}^{\beta_{jk}}}$$

where the log odds *β*_*jk*_ uses the following prior distribution:

$${\beta}_{jk}\sim N\left({\mathrm{0,1.82}}^2\right)$$

The mean and variance specified in the normal distribution above is roughly equivalent to a *Beta* (1, 1) distribution on the response rate *P*_*jk*_, which is widely selected as non-informative prior in the beta-binomial distribution scenario.

#### Bayesian hierarchical model

Motivated from previous work [31], in the *Bayesian hierarchical model*, the prior distribution of the response rate in each subgroup after receiving treatment *k* follows a beta distribution with hyperparameters (*mP*_*k*_, *m*(1 − *P*_*k*_)). This prior distribution allows response rates to borrow information among all subgroups within treatment *k*. Here, *m* is a constant that represents how strong our prior belief is, before the trial starts, that the treatment response rates *P*_*jk*_ are close to the average response rate *P*_*k*_ in treatment *k*. For example, if *m* is large, it means we are confident that *P*_*jk*_ is very close to *P*_*k*_. In this paper, different *m* values were tested and compared.

The hyperparameter *P*_*k*_ is modeled through a beta distribution with parameters (*α*_*k*_, *β*_*k*_). In this project, since we did not have strong prior knowledge about the average response rate of favorable outcomes, we considered an uninformative uniform prior, which is equivalent to setting *α*_*k*_ and *β*_*k*_ to 1. This uniform prior and can be interpreted as every possible probability of success from 0 to 100% being equally likely.

With this setup, the complete hierarchical model can be written as follows:

$${Y}_{jk}\sim Binomial\left({n}_{jk},{P}_{jk}\right)$$

$${P}_{jk}\sim Beta\left(m{P}_k,m\left(1-{P}_k\right)\right)$$

$${P}_k\sim Beta\left({\alpha}_k,{\beta}_k\right)$$

$${\alpha}_k={\beta}_k=1$$

#### Bayesian hierarchical drift model

Previous models assume the treatment response rates to not change over time. However, this is not always the case in real-life clinical trials. If the response rates changed over time and were not adjusted for properly, severely biased estimates could be obtained thus leading to wrong decisions. Using the same approach described in the REMAP-CAP study [42], the previous Bayesian hierarchical model can be modified to include a drift parameter that accounts for treatment response rates changing over time.

In this model, we consider time points to correspond to interim analyses and the final analysis after completion of the trial. The time-indicating variable *t* is an integer ranging from 1 to *T*, with *T* representing the most recent time point. Each treatment response rate for the most recent time point *P*_*jkT*_ is modeled using the same structure as in the previous hierarchical model. For every previous time point, the response rate is modeled on the log odds scale as the sum of the response rate of the most recent time point and the time effect *θ*_*t*_. The time effect parameters ***θ***_*t*_ are modeled with a first-order normal dynamic linear model (NDLM). The hyper prior of the drift parameter *τ* follows an inverse-gamma distribution. The NDLM allows for borrowing among effects of adjacent time periods, pulling their estimates towards each other, and can robustly handle different trends over time. The borrowing is controlled by the drift parameter *τ*. The full model can be summarized below, for the last time point T,

$${Y}_{jkT}\sim Binomial\left({n}_{jkT},{P}_{jkT}\right)$$

$${P}_{jkT}\sim Beta\left(m{P}_k,m\left(1-{P}_k\right)\right)$$

$${\theta}_T=0$$

For all *t* < *T*

$${Y}_{jkt}\sim Binomial\left({n}_{jkt},{P}_{jkt}\right)$$

$$logit\left({P}_{j,k,t}\right)= logit\left({P}_{jkT}\right)+{\theta}_t$$

$${\theta}_{t-1}\sim N\left({\theta}_t,\tau \right)$$

$$\tau \sim InvGamma\left(\mathrm{0.25,0.1}\right)$$

### Bayesian quantities of interest

#### Posterior probability of treatment difference

For each treatment, *k* = *EVT*, *MM*, the posterior probability of treatment difference *P*(*P*_*jk*_ − *P*_*jk*_ > 0) within a subgroup *j* can be understood as the posterior probability that one treatment *k* is superior to another treatment *k*^′^.

After samples are drawn from each respective posterior distribution, the probabilities of treatment difference are calculated as the proportion of posterior samples where respectively either *P*_*j*, *EVT*_ − *P*_*j*, *MM*_ or *P*_*j*, *MM*_ − *P*_*j*, *EVT*_ is greater than 0.

#### Odds ratio

For each subgroup, we calculate the posterior odds ratio of the probability of obtaining a favorable outcome response between two treatments as such:

$$O{R}_j=\frac{\frac{P_{jMM}}{1-{P}_{jMM}\ }}{\frac{P_{jEVT}}{1-{P}_{jEVT}}}$$

### Study design and patient accrual

The motivating study envisions a trial which recruits and follows subjects for 4 years with three interim analyses and one final analysis. The first interim is scheduled to occur after 2500 participants have enrolled into the trial. Subsequent interims will be conducted after every additional 2500 participants are enrolled and will continue until a total of 10,000 participants are enrolled. Since interim analyses are defined by participants enrolled, the timing of the interims is random and will depend on the rate at which participants accrue to the trial. Overall, since we expect to enroll 10,000 participants in 4 years, an average of 52 participants have to be enrolled per week. Here, we assume the patient accrual will follow a Poisson distribution with parameter 52.

We considered three different study designs:

1. A fixed allocation design in which patients are always allocated to the two treatment arms in a 1:1 ratio. No interim analysis will be performed during the trial process.
2. A response-adaptive randomization (RAR) design that updates allocation to favor the more promising treatment at each interim based on the Bayesian quantities of interest.
3. A modified RAR design that finds a compromise between the 1:1 and the pure RAR allocation ratios, named “RARCOMP”.

For both RAR and RARCOMP designs, three interim analyses and one final analysis were performed as described above. Details about the adaptive randomization schemes will be explained in the next section.

### Patient allocation in adaptive designs

**Fig. 2:**
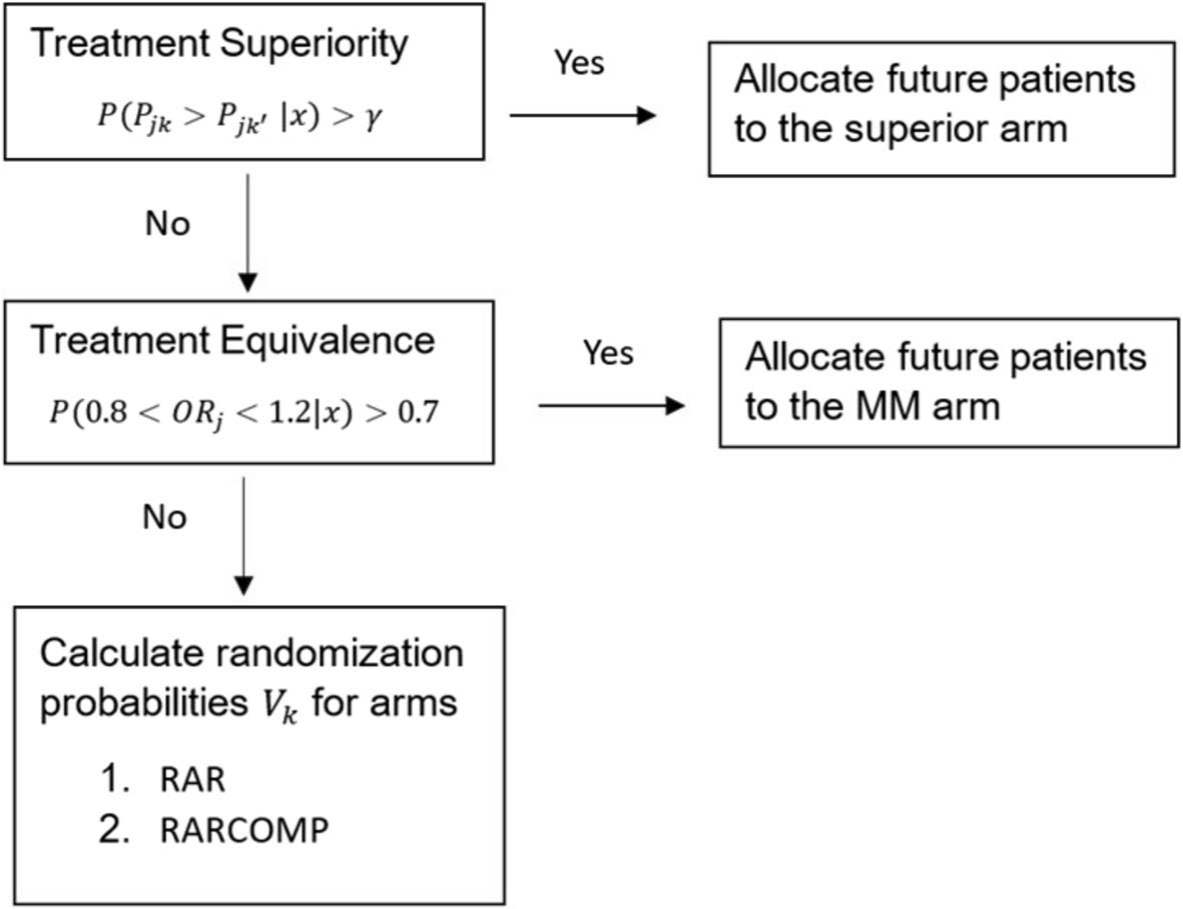
Adaptive patient allocation flowchart

Adaptive randomization will begin right after the trial starts, using within subgroup prior information, and is performed at each interim, with the goal to allocate more subjects to the treatment that appears to be more promising. Bayesian quantities of interest discussed above were used to guide decisions. The patient allocation flowchart in Fig. 2 briefly summarizes how patients were allocated in a single clinical trial. The posterior response rates for both treatment arms were compared for each patient subgroup. If a superiority criterion was satisfied for any subgroup, all future patients would be allocated to the superior arm for that patient subgroup. Equivalence of the two treatments were tested if the superiority criterion was not met. An establishment of equivalence would lead to all future patients being allocated to the MM treatment for lower cost. If neither superiority nor equivalence were established, the patient allocation rates for the two treatments would be calculated using prespecified allocation schemes. Details about patient allocation will be provided in this section.

#### Allocation for expected success

Patient randomization information may change at each interim analysis due to expected success and allocate all future participants to the superior treatment if the following criteria is satisfied, where *k* and *k*^′^ represent different treatments:

$$P\left({P}_{jk}>{P}_{j{k}^{\prime }}\right)>\gamma$$

The value of *γ* was obtained based on simulation and controlled for the two-sided overall type 1 error to be close to 0.05. It varies for different *m* values and for different randomization schemes (the “Type 1 error calibration” section).

#### Allocation for equivalence (effectively MM should be used)

During each interim, if the expected success condition is not met, the trial may change patient randomization due to equivalence and allocate all future participants to the MM treatment, since it is a less expensive treatment option. Equivalence is established if the following criterion is satisfied:

$$P\left(0.8<O{R}_j<1.2\right)>0.7$$

The utilized boundaries for odds ratios in the above criterion have been traditionally used in bioequivalence studies and were selected based on this fact.

#### Allocation when no success or equivalence is met

If neither superiority nor equivalence is identified, the patient allocation rates are calculated based on prespecified randomization schemes. They are (1) the common RAR allocation scheme and (2) RARCOMP — the modified RAR scheme.

For RAR, the probability *V*_*jk*_ of the next participant being allocated to treatment *k* in subgroup *j* was calculated such that it satisfies the formula shown below:

$${V}_{jk RAR}\propto \sqrt{P\left({P}_{jk}-{P}_{j{k}^{\prime }}>0\right)\frac{Var\left({P}_{jk}\right)}{n_{jk}+1}}$$

where *Var*(*P*_*jk*_) are the posterior variances of the mean response rates, *n*_*jk*_ is the current number of participants in subgroup *j* assigned to treatment *k*, and *k*^′^ being the treatment arm other than *k*. The randomization probabilities for treatments will be updated once at each interim.

For the fixed 1:1 allocation, these probabilities were both 0.5.

$${V}_{jkfixed}={V}_{jkfixed}^{\prime }=\frac{1}{K}=0.5$$

RARCOMP represents a tradeoff between RAR and the fixed 1:1 allocation, where the allocation rate for treatment *k* is then the average of *V*_*k*_ and 0.5. The allocation rate for the new RAR compromise patient allocation scheme can thus be written as:

$${V_{jk}}_{compromise}=\frac{V_{jk RAR}+{V}_{jk fixed}}{2}$$

#### Initial allocation in adaptive designs

In the RAR and RARCOMP schemes, prior knowledge provided by the experts was used to inform patient allocation within subgroups at the start of the trial. This was done as follows: Let ${P}_{jk}^0$ be the current understanding of the rate of favorable outcome for treatment *k* in subgroup *j*. Let ${n}_{jk}^0$ be the prior sample size for the treatment *k* in subgroup *j*. Before the trial starts, create a pseudo-dataset with ${n}_{jk}={n}_{jk}^0=10$ observations in each subgroup-treatment combination and a response of ${Y}_{jk}={P}_{jk}^0\times {n}_{jk}^0$. Sample size for the pseudo-dataset was chosen to be 10, so that previous information was incorporated in the design but not too overpowering to bias the estimates. Based on this data, calculate posterior quantities of interest and follow the allocation rules of the study protocol. Prior knowledge about ${P}_{jk}^0$ utilized in this trial is shown in Table 1.

**Table 1:** Prior information to calculate the patient allocation before trial starts

Subgroup	***P***_***MM***_	***P***_***EVT***_
Large Core Only	0.10	0.25
Mild Deficit Only	0.70	0.84
Distal Occlusion Only	0.35	0.55
Distal Occlusion + Large core	0.25	0.45
Distal Occlusion + Mild Deficit	0.75	0.85

### Simulation study

In this paper, we investigated and compared scenarios where the *m* value varies from 1 to 30 (*m* ∈ {1, 10, 20, 30}) for Bayesian hierarchical models.

#### Simulating data without drift effect

We simulated 10,000 clinical trial studies to investigate the model operating characteristics for each design. In order to study design performance, five simulation scenarios were considered: (1) one “equal” scenario in which the favorable outcome rates of MM and EVT are simulated to be the same (averaging across MM and EVT treatment for each subgroup), (2) an “expected” scenario where the favorable outcome response rates in EVT is simulated to be higher than in MM based on the previous knowledge, (3) a “reverse” scenario where the favorable outcome response rate in MM is simulated to be higher than in EVT, (4) an “extreme” case where the favorable outcome response rate in EVT is simulated to be much higher than in MM, and (5) a scenario in which “single subgroup” is better in EVT while the two treatments are the same for the rest of the subgroups. Details about the four scenarios are shown in Table 2.

**Table 2:** A summary of five simulation scenarios without drift effect

Subgroup	Equal		Expected		Reversed		Extreme EVT		Single subgroup	
	*P*_*MM*_	*P*_*EVT*_	*P*_*MM*_	*P*_*EVT*_	*P*_*MM*_	*P*_*EVT*_	*P*_*MM*_	*P*_*EVT*_	*P*_*MM*_	*P*_*EVT*_
Large Core Only	0.1	0.1	0.1	0.25	0.25	0.1	0.1	0.30	0.25	0.45
Mild Deficit Only	0.7	0.7	0.7	0.84	0.84	0.7	0.7	0.89	0.84	0.84
Distal Occlusion Only	0.35	0.35	0.35	0.55	0.55	0.35	0.35	0.60	0.55	0.55
Distal Occlusion + Large core	0.25	0.25	0.25	0.45	0.45	0.25	0.25	0.50	0.45	0.45
Distal Occlusion + Mild Deficit	0.75	0.75	0.75	0.85	0.85	0.75	0.75	0.90	0.85	0.85

#### Simulating data with drift effect

Similarly, we also simulated all five scenarios when a drift effect was present in the data. To achieve this, the true response rates for the last time point *P*_*jkT*_ were chosen to be the same as the values in Table 2. However, the log odds of response rates for previous time points were set to decrease linearly over time. Under this simulation setup, response rates in earlier stages of the trial were higher than in the later stages. The simulated response rates for each time point are summarized in Table 3.

**Table 3:** Linear time effects for response rates used in simulation studies

	*t* = 1	*t* = 2	*t* = 3	*t* = 4
*θ*_*t*_	0.75	0.5	0.25	0
*logit*(*P*_*jkt*_)	*logit*(*P*_*jkT*_) + 0.75	*logit*(*P*_*jkT*_) + 0.5	*logit*(*P*_*jkT*_) + 0.25	*logit*(*P*_*jkT*_) + 0

*t* represents each interim analysis, *θ*_*t*_ represents time effects at different time points

#### Model operating characteristic evaluation

Bayesian hierarchical modeling was performed using the R (version 3.5.3) package “Nimble” [53] (version 0.9.0) (code provided in Additional file 1: Appendix (c) in the supporting material). The results of the adaptive designs were then compared with two versions of fixed 1:1 allocation designs: one using the Bayesian hierarchical model fit in Nimble and another using the independent model fit in the Fixed and Adaptive Clinical Trial Simulator (FACTS) (Berry & Sanil, 2010) software [54] (version 6.3), having no interims. The independent model fitted in FACTS is served as the standard design; however, it is limited.

The type 1 error for a two-sided test was obtained from the “Equal” scenario. For models not accounting for drift in non-drift scenarios, it was calibrated to the 0.05 level by adjusting *γ* in designs simulated in R and NIMBLE. For drift models, the same thresholds obtained for non-drift models were used and type 1 error was not recalibrated. Using these *γ* values, statistical power was then evaluated in the remaining scenarios. Generally, the type 1 error was calculated as the proportion of simulations in which either EVT is superior, or MM is superior under the true scenario that EVT and MM have the same response rate; while power was calculated as the proportion of simulations which correctly exhibit superiority of either treatment under scenario “Expected,” “Reversed,” and “Extreme EVT.”

## Results

### Type 1 error calibration

For non-drift models, overall type 1 error was successfully controlled at the 0.05 level in all simulated non-drift scenarios. The required *γ* thresholds tended to decrease with *m* when employing a fixed allocation scheme but remained stable at approximately 0.995 when employing the response-adaptive designs. The simulation based overall type 1 errors for all scenarios as well as their corresponding *γ* values are provided in the Additional file 1: Appendix (a) in the supporting material.

### Bayesian hierarchical model on data without drift effect

**Fig. 3:**
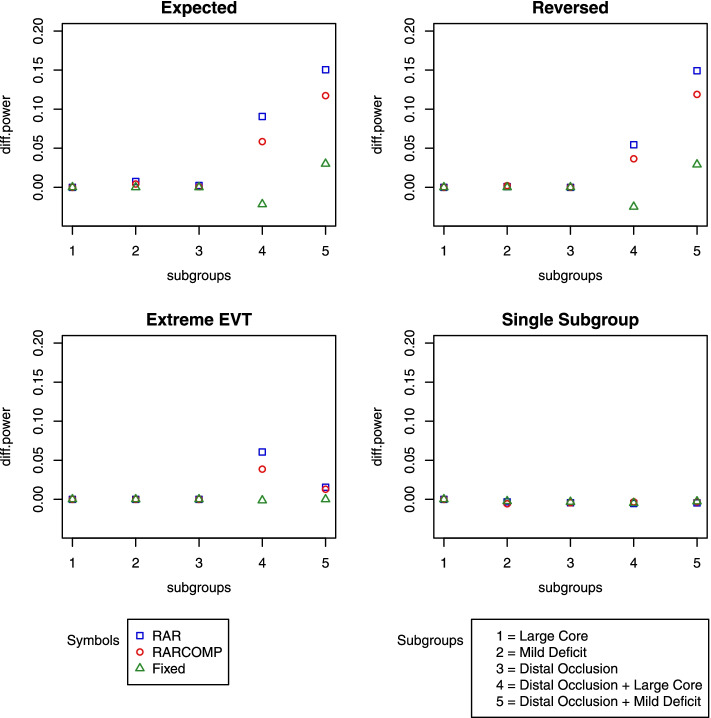
Power difference among three adaptive design schemes relative to the fixed design when *m* = 1

Statistical power for different randomization schemes was compared after calibrating the overall type 1 error at the 0.05 level. Figure 3 shows a comparison among three randomization schemes using Bayesian hierarchical model fit data that does not have a time drift effect under all alternative scenarios when *m* value is set to be 1. With the *y*-axis being the difference in power between the fixed design (with independent model) and the three adaptive designs with various randomization schemes respectively (with hierarchical models), for example, one of the *y* value could be Power_fixed independent model_ − Power_adaptive Bayesian hierarchical_. Since an equal allocation of patients in the two-arm setting provides higher power, treating the independent fixed model as a reference, a smaller *y* value in Fig. 3 indicates a higher statistical power. When *m* = 1, in all scenarios, among all randomization schemes, fixed allocation appeared to have the highest statistical power. This power difference was not strong for the first three subgroups as the sample sizes in those subgroups were large. However, the differences were extremely obvious for subgroups “Distal Occlusion + Large Core” and “Distal Occlusion + Mild Deficit” in scenarios “Expected”, “Reversed,” and “Extreme EVT” due to small sample sizes. RAR randomization scheme appeared to have the lowest statistical power as the *y* values for RAR tend to be the highest among the three schemes. RARCOMP scheme provided power higher than RAR, but lower than fixed 1:1 allocation rule.

**Fig. 4:**
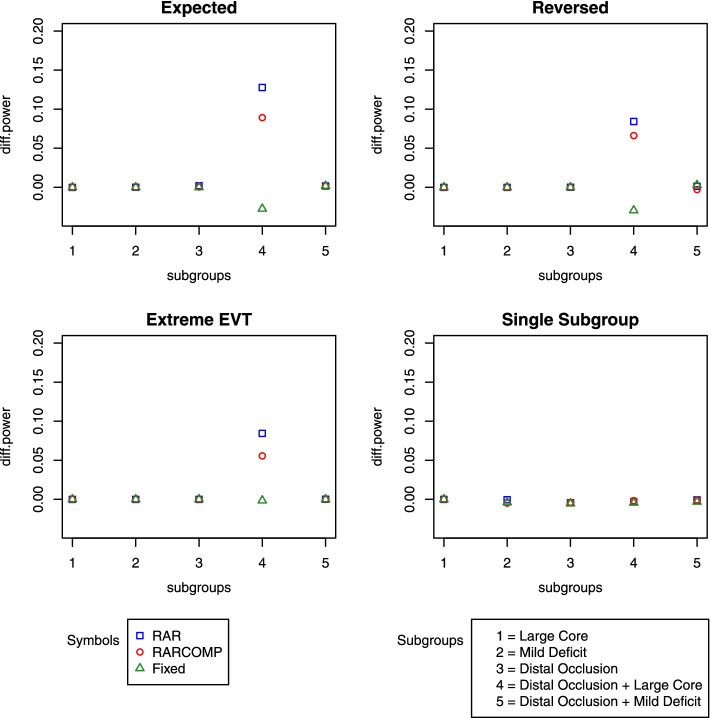
Power difference among three adaptive design schemes relative to the fixed design when *m* = 30

Increasing the *m* value from 1 to 30, the statistical power increased for all randomization schemes in all scenarios for subgroup “Distal Occlusion + Mild Deficit” as the *y* values for that subgroup dropped to 0 for all schemes. However, the power was decreased for subgroup “Distal Occlusion + Large core” (Fig. 4). In this subgroup, we can reach the same conclusion as before that the fixed 1:1 allocation obtained the highest power followed by RARCOMP allocation scheme. The power obtained from the RAR scheme was the lowest among all three schemes.

The inconsistent behavior in statistical power between the last two subgroups was caused by increased estimation bias when increasing the m value in the model. A brief demonstration of how power changes for scenario “Expected” can be found in Additional file 1: Appendix (b) in the supporting material.

**Fig. 5:**
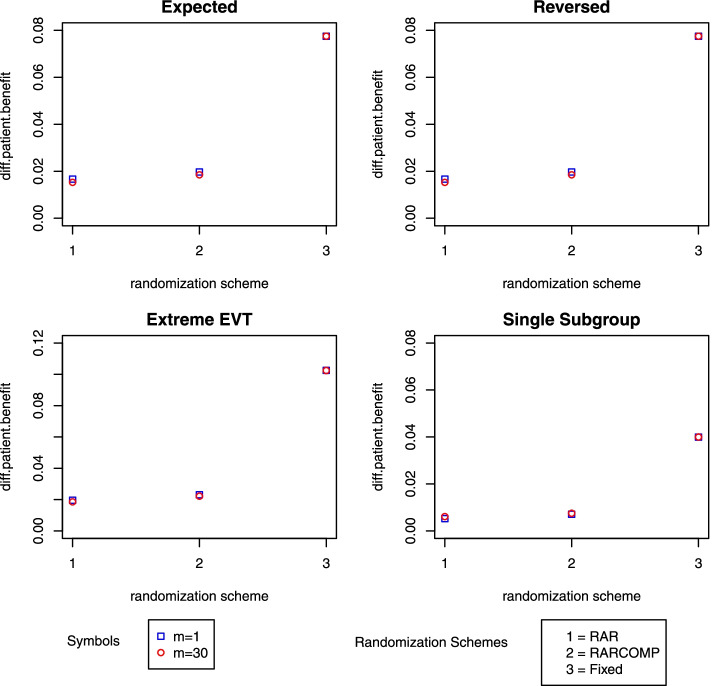
Patient benefit comparison for three randomization schemes

One of the benefits of using adaptive designs is to allocate more patients to the better performed treatment, thus improving the patient benefit within the trial. Figure 5 shows the patient benefit comparison among the three randomization schemes stratified by m value. The *y-*axis represents the difference between the hypothetical subjects’ proportion with good outcomes and the observed subject proportion with good outcomes, with the former being the proportion of subjects that would experience a good outcome in a perfect world, where all subjects are always allocated to the treatment arm with the highest success rate, and the latter being the proportion of observed good outcomes in the simulated trials. In this way, a smaller *y* value indicates higher patient benefits. In Fig. 5, the RAR scheme obtained the highest patient benefit, which was closely followed by the RARCOMP scheme. The fixed allocation scheme achieved the lowest patient benefit among all three schemes. Comparing *m*=1 to *m*=30 alone, although the differences were small, *m*=30 obtained higher patient benefit under all schemes for most of the scenarios.

In summary, the RARCOMP randomization scheme has shown to improve statistical power compared to the regular RAR scheme, without compromising too much patient benefit. Also, increasing *m* led to a higher statistical power but also more biased estimates. Since *m*=30 provided the best performance (higher power, higher patient benefit with moderately biased estimates), in this paper, we will focus on the model performance under *m*=30 setup.

### Bayesian hierarchical model on data with drift effect

**Fig. 6:**
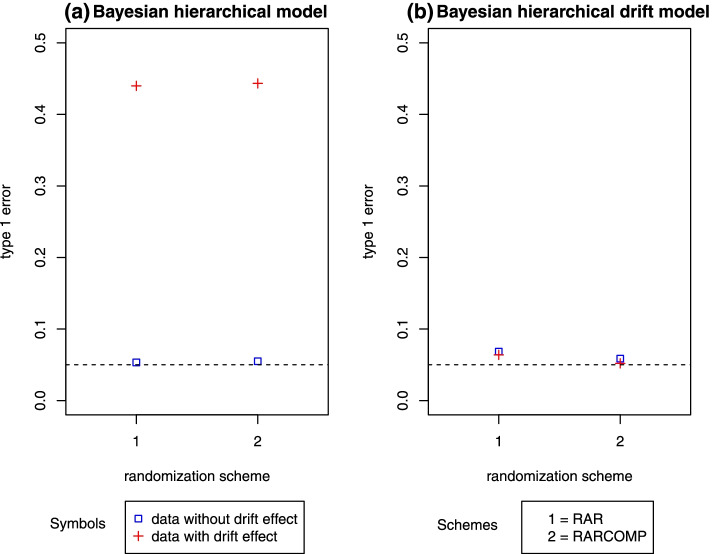
Type 1 error comparison for the Bayesian hierarchical model and the Bayesian hierarchical drift model

The previous results compare the three randomization schemes when Bayesian hierarchical models were fit to the data without a time drift effect. When fitting the same model to data in which the response rates changed over time and using response-adaptive randomization instead of a fixed 1:1 allocation scheme, a huge inflation in type 1 error was observed (Fig. 6a).

### Bayesian hierarchical drift model on data with drift effect

Fitting our Bayesian hierarchical drift model to data in which the response rates changed over time using response-adaptive randomization and the same thresholds as the model not accounting for drift, type 1 error slightly increased but was well controlled below 0.07 for both randomization schemes (Fig. 6b).

**Fig. 7:**
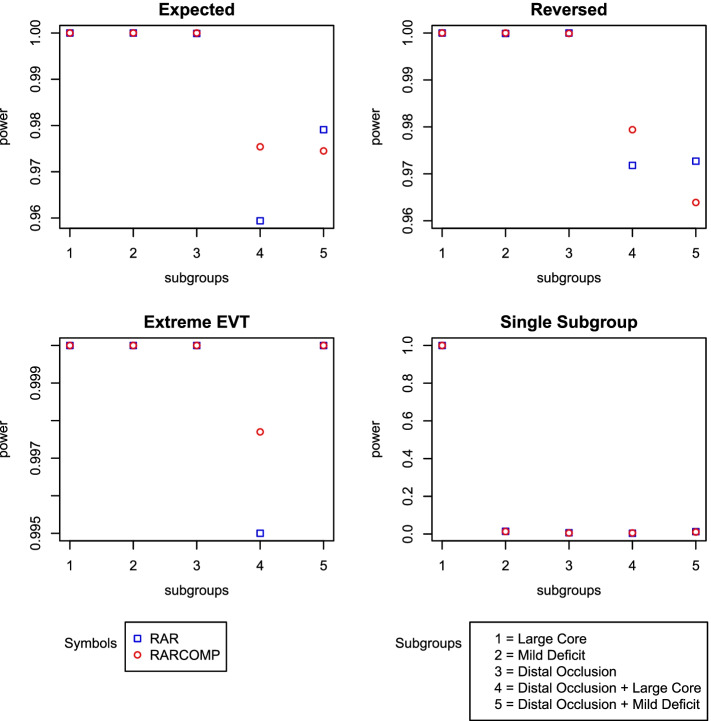
Power plots: fit Bayesian hierarchical drift model to linear time effect drift data

In addition to the well-controlled type 1 error, the Bayesian hierarchical drift model also established a very high performance. High statistical power was observed for all alternative scenarios for both randomization schemes. RARCOMP appeared to have higher power than RAR in subgroup “Distal Occlusion + Large core.” Although the differences were small, RAR showed higher power for “Distal Occlusion + Mild Deficit” in scenarios “Expected” and “Reversed” compared with RARCOMP (Fig. 7).

**Fig. 8:**
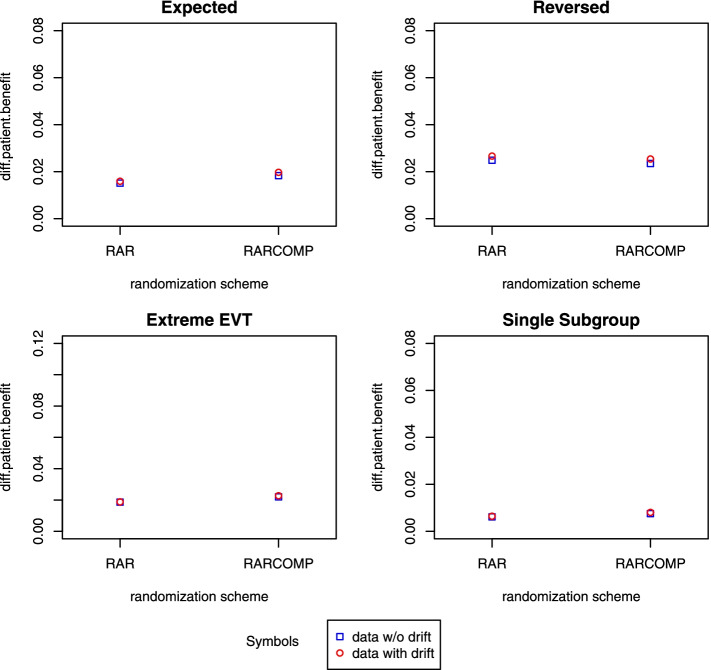
Patient benefit when fitting the Bayesian drift model

Patient benefit was also compared between RAR and RARCOMP scheme. In Fig. 8, a red circle was used to indicate the scenario when the drift model was fitted to the linear drift effect data. The *y*-axis represents the differences in patient benefit between the hypothetical proportion of patients obtaining good outcomes and the observed proportion of patients with good outcomes, a smaller *y*-axis value indicating a higher patient benefit. RAR and RARCOMP both obtained very high patient benefit, with the RAR scheme achieving slightly higher values.

### Bayesian hierarchical drift model on data without drift effect

**Fig. 9:**
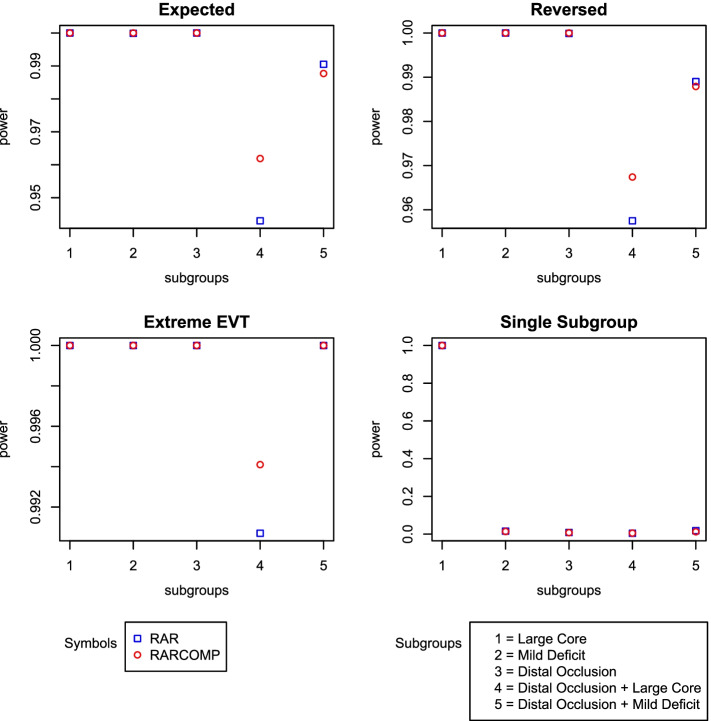
Power plots: fit Bayesian hierarchical drift model to data without a linear effect

We have shown previously that the Bayesian hierarchical drift model handles response rates drift over time well when the drift effect is linear across time. However, to be a promising and robust model, the model still needs to perform well in situations where the time drift effect is absent. Figure 9 shows the power remained very high even when our drift model was fitted to a dataset that does not have a linear time effect. Comparing RAR and RARCOMP, the RARCOMP allocation scheme achieved higher statistical power especially in the subgroup “Distal Occlusion + Large Core.”

Patient benefit was also evaluated for this setup. In Fig. 8, a blue box was used to indicate the scenario when the drift model was fitted to the data without a drift effect. Comparing the patient benefit when fitting the same model to both data with linear drift effect (red circle in Fig. 8) and to data without linear drift effect (blue box in Fig. 8), values were very similar, suggesting that this model was very robust against whether or not a linear time effect was present in the data.

## Discussion

Our simulation studies have shown that the RARCOMP scheme can provide high statistical power while maintaining high patient benefit in all simulated scenarios. However, the use of RAR in two-arm studies has been controversial [39, 46, 55]. Previous work has shown using RAR in two-arm trials without careful planning and calibration could result in biased estimates and might even lead to wrong conclusions. In addition to its ability to balance statistical power and patient benefit, the RARCOMP scheme could help to mitigate this issue. The fact that it averages allocation ratios between the naïve RAR and the fixed 1:1 randomization prevents the allocation process from creating highly unbalanced sample sizes between the two treatments and makes it more robust to RAR bias.

As response-adaptive designs are more susceptible to drift effects [55], in this paper, we also incorporated a drift parameter in the Bayesian model to account for response rate drift over time. Simulation results demonstrated our drift model can accurately estimate linear trend drift effects over time and account for these changes when comparing treatments. Moreover, even when time effects were absent in the data, our drift model still performed well and retained high statistical power. In combination with the fact that the NDLM component used to estimate time effects is able to flexibly model different shapes, our results suggest that this approach can be robustly applied in many clinical trial scenarios.

Current simulation results have confirmed our drift model works well on data with linear drift effect. More work needs to be done to confirm the drift model also maintains high performance in other situations. However, since we used non-informative priors on the drift effect *τ*, as long as the change in the response rates are not dramatic during a period of time, it is safe to guess our model could perform well even when the time effects are nonlinear.

In conclusion, with the ability to have high power and good patient benefit and to account for population drift, our design using the Bayesian hierarchical drift model with the RARCOMP scheme is a promising choice for adaptive trials. This article introduces the novel idea of combining the traditional RAR scheme and fixed 1:1 allocation to provide a nice balance between them. Our design is robust against both severely unbalanced allocation and drift over time.

## Supplementary Information

**Additional file 1: Appendix a.** A Summary of γ and the controlled type 1 error rates for different scenarios. **Appendix b.** Demonstration Example of power change when increasing m. **Appendix c.** Bayesian Hierarchical Model Nimble Code. **Appendix d.** Bayesian Hierarchical Drift Model Nimble Code.

## Data Availability

No real-world data was collected in this study. Nimble code to perform model fitting is provided in Additional file 1: Appendix (c) in the supporting material.

## Abbreviations

RAR: Response-adaptive randomization
MAMS: Multi-arm, multi-stage
EVT: Endovascular thrombectomy
MM: Medical management
NOSI: Notice of special interest
STEP-STONE: StrokeNet ThrombEctomy Platform - STarting with OptimizatioN of Eligibility
NDLM: Normal dynamic linear model
RARCOMP: An innovative RAR allocation scheme
mRS: Modified Rankin scale
FACTS: Fixed and Adaptive Clinical Trials Simulator
