# Supplementary material for: Optimizing a Bayesian hierarchical adaptive platform trial design for stroke patients
Source: Trials. 2022 Sep 6;23:754. doi: 10.1186/s13063-022-06664-4 (PMC9446515; doi:10.1186/s13063-022-06664-4)
Supplement: Supplementary file 1 — Additional file 1: Appendix a. A Summary of γ and the controlled type 1 error rates for different scenarios. Appendix b. Demonstration Example of power change when increasing m. Appendix c. Bayesian Hierarchical Model Nimble Code. Appendix d. Bayesian Hierarchical Drift Model Nimble Code. [file 13063_2022_6664_MOESM1_ESM.docx]

**Appendix**

1. **A Summary of** $\boldsymbol{\gamma}$ **and the controlled type 1 error rates for different scenarios**

|  | $\boldsymbol{\gamma}$ **Value** | **Type 1 Error** |
| --- | --- | --- |
| **Fixed Allocation** |  |  |
| $m=1$ | 0.9955 | 0.049 |
| $m=10$ | 0.9948 | 0.051 |
| $m=20$ | 0.994 | 0.051 |
| $m=30$ | 0.994 | 0.052 |
| **RAR Allocation** |  |  |
| $m=1$ | 0.995 | 0.051 |
| $m=10$ | 0.995 | 0.05 |
| $m=20$ | 0.995 | 0.049 |
| $m=30$ | 0.995 | 0.053 |
| **RAR Compromise Allocation** |  |  |
| $m=1$ | 0.9948 | 0.051 |
| $m=10$ | 0.9948 | 0.053 |
| $m=20$ | 0.9948 | 0.052 |
| $m=30$ | 0.995 | 0.049 |

1. **Demonstration Example of power change when increasing m**

Take Scenario “Expected” and RAR randomization scheme for example, the visualization of the posterior distribution of response rates for that setup was shown in figure appendix (1). In these plots, the true response rates that were simulated are marked as red stars. The estimated posterior means were marked as black dots and the estimated 95% credible intervals were displayed as blue error bars. When m equals 1, the posterior means were all very close to the true values of the response rates. After m value was increased to 30, all the subpopulation response rates were pulled towards the overall mean of the treatment response rate. This caused slightly biased estimates. For subgroup “Distal Occlusion + Mild Deficit”, this led to the posterior distributions of the response rates in the MM arm and the EVT arm to be pulled away from each other. The distance between the credible intervals is larger when m equals 30, compared to when m equals 1. As a result, the power in detecting the difference between MM and EVT has increased in this subgroup. The same effect happens in other subgroups, except for subgroup “Distal Occlusion + Large core”. In that subgroup, the posterior distributions of the response rates were pulled towards each other. When comparing m equals 1 to m equals 30, the distance between the credible intervals of MM and EVT decreased which led to a decreased ability in detecting the difference between MM and EVT in this subgroup.


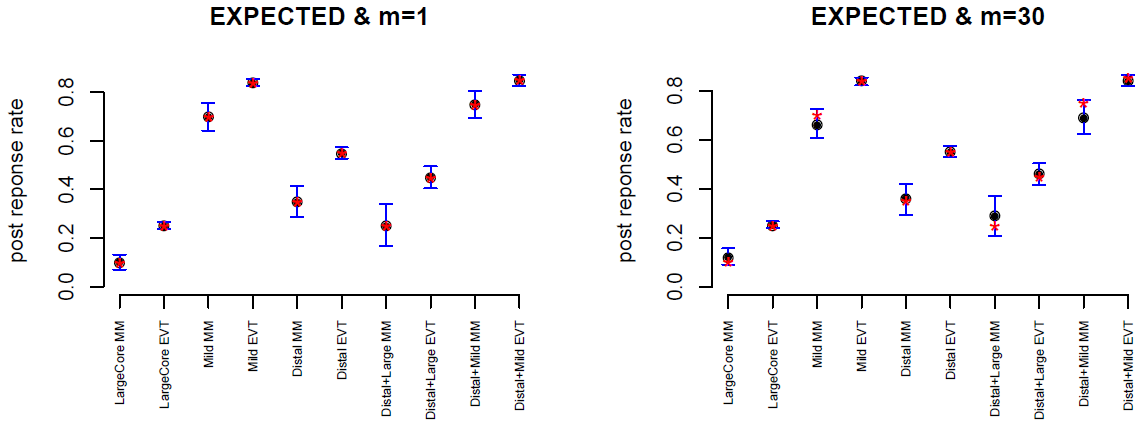


**Figure appendix (1)**. An example of biased posterior estimates.

I = 95% credible interval * = true rate • = posterior point estimate

1. **Bayesian Hierarchical Model Nimble Code**

1. **Bayesian Hierarchical Drift Model Nimble Code**

#------------------------- openbugs model -----------------------------#

Byrons_Model<-nimbleCode(

{

for(j in 1:g)

{

for(k in 1:2)

{

Y[j,k]~dbinom(prob = P[j,k],size = n[j,k])

P[j,k]~dbeta(alpha[k],beta[k])

}

}

for(k in 1:2)

{

alpha[k]<-m0*PP[k]

beta[k]<-m0*(1-PP[k])

PP[k]~dbeta(alpha0[k],beta0[k])

}

for(j in 1:g)

{

C[j]<-P[j,1]-P[j,2]

OR[j]<-(P[j,2]/(1-P[j,2]))/(P[j,1]/(1-P[j,1]))

ProbMM[j]<-step(C[j])

ProbEVT[j]<-1-step(C[j])

lower_OR[j]<- 0.8 - OR[j]

upper_OR[j]<- OR[j] - 1.2

# Below_OR is 1 if OR is below equivalence lower bound

Below_OR[j]<-step(lower_OR[j])

# Above_OR is 1 if OR is above equivalence upper bound

Above_OR[j]<-step(upper_OR[j])

#1-Below_OR[j] implies OR is greater than .8

#1-Above_OR[j] implies OR is less than 1.2

#If their product is 1, then we are in equivalence range

Equivalence[j]<-(1-Below_OR[j])*(1-Above_OR[j])

}

}

)

Drift_Model<-nimbleCode(

{

# theta[TT] <- 0 # already assigned by initial values

for(j in 1:g)

{

for(k in 1:2)

{ # TT is the last time point

P[j,k,TT]~dbeta(alpha[k],beta[k])

Y[j,k,TT]~dbinom(prob = P[j,k,TT],size = n[j,k,TT])

for(t in 1:TM1)

{

Y[j,k,t]~dbinom(prob = P[j,k,t],size = n[j,k,t])

logit(P[j,k,t]) <- logit(P[j,k,TT]) + theta[t]

}

}

}

for(t in 1:TM1)

{

theta[t] ~ dnorm(theta[t+1],tau)

}

tau ~ dgamma(0.25,0.1)

for(k in 1:2)

{

alpha[k]<-m0*PP[k]

beta[k]<-m0*(1-PP[k])

PP[k]~dbeta(alpha0[k],beta0[k])

}

for(j in 1:g)

{

C[j]<-P[j,1,TT]-P[j,2,TT]

OR[j]<-(P[j,2,TT]/(1-P[j,2,TT]))/(P[j,1,TT]/(1-P[j,1,TT]))

ProbMM[j]<-step(C[j])

ProbEVT[j]<-1-step(C[j])

lower_OR[j]<- 0.8 - OR[j]

upper_OR[j]<- OR[j] - 1.2

# Below_OR is 1 if OR is below equivalence lower bound

Below_OR[j]<-step(lower_OR[j])

# Above_OR is 1 if OR is above equivalence upper bound

Above_OR[j]<-step(upper_OR[j])

#1-Below_OR[j] implies OR is greater than .8

#1-Above_OR[j] implies OR is less than 1.2

#If their product is 1, then we are in equivalence range

Equivalence[j]<-(1-Below_OR[j])*(1-Above_OR[j])

}

}

)
